# Supplementary material for: Nutritional Considerations for Bladder Storage Conditions in Adult Females
Source: Int J Environ Res Public Health. 2023 Oct 3;20(19):6879. doi: 10.3390/ijerph20196879 (PMC10573006; doi:10.3390/ijerph20196879)
Supplement: Supplementary file 1 [file ijerph-20-06879-s001.zip › ijerph-2601779-supplementary.pdf]

Supplemental Tables: Nutritional Considerations for Bladder Storage Conditions in Adult Females

Table S1: List of Urological Association Websites Accessed for Relevant Clinical Guidelines Stratified by Continent

		Guideline Found but Not in English	Guideline Found but Not in Date Range	No Guideline Found
**GLOBAL**				
ESSIC (International Society for the Study of Bladder Pain Syndrome)			X	
International Association for the Study of Pain				X
International Federation of Gynecology and Obstetrics				X
**AFRICA**				
African Federation of Obstetricians and Gynaecologists				X
College of Urologists of South Africa			X	
Pan African Urological Association			X	
South African Society of Obstetricians and Gynaecologists				X
South African Urological Association			X	
**ASIA**				
Afghan Urology Association				X
Arab Association of Urology				X
Asia-Pacific Urogynecology Association				X
Chinese Urology Association				X
Egyptian Urological Association				X
Iranian Urological Association				X
Israel Urology Association				X
Japanese Urological Association		X		
Korean Association of Urogenital Tract Infection and Inflammation				X
Korean Urological Association				X
Neurogenic Bladder Society			X	
Taiwan Association of Obstetrics and Gynecology				X
Taiwan Urogynecology Association				X
Urological Association of Asia	X			X
URPSSI (India Urology Association)				X
**AUSTRALIA and OCEANIA**				
National Association of Specialist Obstetricians and Gynaecologists				X
NZ Nocturia Guideline Committee			X	
Royal Australian and New Zealand College of Obstetricians and Gynaecologists				X
**EUROPE**				
Austrian Guideline Pelvic Pain Syndrome				X
European Society of Gynecology				X
European Society of Obstetricians and Gynecologists				X
European Urogynaecological Association				X
German group of experts				X
German Society of Urology		X		
National Association of Specialist Obstetricians and Gynaecologists				X
Nederlandse Vereniging voor Urologie		X		
Spanish Urological Association		X		

Table S1: List of Urological Association Websites Accessed for Relevant Clinical Guidelines Stratified by Continent

	Guideline Found but Not in English	Guideline Found but Not in Date Range	No Guideline Found
**NORTH AMERICA**			
Sociedad Mexicana de Urologia			X
**SOUTH AMERICA**			
Confederación Americana de Urología	X		
Ecuadorian Society of Urology			X
Ibero-American Society of Neurourology and Urogynecology			X
Latin American Federation of Obstetrics and Gynecology Societies			X
Sociedad Argentina de Urologia			X
Sociedad Colombiana de Urologia			X
Société Internationale d'Urologie			X
Urological Society of Brazil			X
Urological Society of Chile			X
Urological Society of Paraguay			X
Urological Society of Peru			X
Urological Society of Venezuela			X

Table S2: Agree II Quality Analysis Template

**DOMAIN 1. SCOPE AND PURPOSE**						
1. The overall objective(s) of the guideline is (are) specifically described.						
1	2	3	4	5	6	7
Strongly Disagree						Strongly Agree
Comments						
2. The health question(s) covered by the guideline is (are) specifically described.						
1	2	3	4	5	6	7
Strongly Disagree						Strongly Agree
Comments						
3. The population (patients, public, etc.) to whom the guideline is meant to apply is specifically described.						
1	2	3	4	5	6	7
Strongly Disagree						Strongly Agree
Comments						
**DOMAIN 2. STAKEHOLDER ENGAGEMENT**						
4. The guideline development group includes individuals from all relevant professional groups.						
1	2	3	4	5	6	7
Strongly Disagree						Strongly Agree
Comments						
5. The views and preferences of the target population (patients, public, etc.) have been sought.						
1	2	3	4	5	6	7
Strongly Disagree						Strongly Agree
Comments						
6. The target users of the guideline are clearly defined.						
1	2	3	4	5	6	7
Strongly Disagree						Strongly Agree
Comments						
**DOMAIN 3. RIGOUR OF DEVELOPMENT**						
7. Systematic methods were used to search for evidence.						
1	2	3	4	5	6	7
Strongly Disagree						Strongly Agree
Comments						
8. The criteria for selecting the evidence are clearly described.						
1	2	3	4	5	6	7
Strongly Disagree						Strongly Agree
Comments						
9. The strengths and limitations of the body of evidence are clearly described.						
1	2	3	4	5	6	7
Strongly Disagree						Strongly Agree
Comments						
10. The methods for formulating the recommendations are clearly described.						
1	2	3	4	5	6	7
Strongly Disagree						Strongly Agree
Comments						
11. The health benefits, side effects, and risks have been considered in formulating the recommendations The health benefits, side effects, and risks have been considered in formulating the recommendations.						
1	2	3	4	5	6	7
Strongly Disagree						Strongly Agree
Comments						
12. There is an explicit link between the recommendations and the supporting evidence.						
1	2	3	4	5	6	7
Strongly Disagree						Strongly Agree
Comments						
13. The guideline has been externally reviewed by experts prior to its publication.						
1	2	3	4	5	6	7
Strongly Disagree						Strongly Agree
Comments						
14. A procedure for updating the guideline is provided.						
1	2	3	4	5	6	7
Strongly Disagree						Strongly Agree
Comments						
**DOMAIN 4. CLARITY OF PRESENTATION**						
15. The recommendations are specific and unambiguous.						
1	2	3	4	5	6	7
Strongly Disagree						Strongly Agree
Comments						

16. The different options for management of the condition or health issue are clearly presented.						
1<br>Strongly Disagree	2	3	4	5	6	7<br>Strongly Agree
Comments						
17. Key recommendations are easily identifiable.						
1<br>Strongly Disagree	2	3	4	5	6	7<br>Strongly Agree
Comments						
**DOMAIN 5. APPLICABILITY**						
18. The guideline describes facilitators and barriers to its application.						
1<br>Strongly Disagree	2	3	4	5	6	7<br>Strongly Agree
Comments						
19. The guideline provides advice and/or tools on how the recommendations can be put into practice.						
1<br>Strongly Disagree	2	3	4	5	6	7<br>Strongly Agree
Comments						
20. The potential resource implications of applying the recommendations have been considered.						
1<br>Strongly Disagree	2	3	4	5	6	7<br>Strongly Agree
Comments						
21. The guideline presents monitoring and/or auditing criteria.						
1<br>Strongly Disagree	2	3	4	5	6	7<br>Strongly Agree
Comments						
**DOMAIN 6. EDITORIAL INDEPENDENCE**						
22. The views of the funding body have not influenced the content of the guideline.						
1<br>Strongly Disagree	2	3	4	5	6	7<br>Strongly Agree
Comments						
23. Competing interests of guideline development group members have been recorded and addressed.						
1<br>Strongly Disagree	2	3	4	5	6	7<br>Strongly Agree
Comments						
**OVERALL GUIDELINE ASSESSMENT**						
Rate the overall quality of this guideline.						
1<br>Strongly Disagree	2	3	4	5	6	7<br>Strongly Agree
I would recommend this guideline for use.						
Yes		Yes, with modifications			No	
Notes						

Supplemental Tables S3-S6 provide the details of the Agree II quality analysis scoring sheets. One scoring sheet was done for each of the four urological conditions. The combined results are presented stratified by organizational authors.

Table S3: **IC/BPS Part 1**—Summary of Agree II Quality Analysis of Clinical Guidelines

	Quality Score				
	**AUA**	**BSUG RCOG**	**CUA**	**EAG**	**EUA**
**DOMAIN 1. SCOPE AND PURPOSE**					
1. The overall objective(s) of the guideline is (are) specifically described.	7	7	3	7	7
2. The health question(s) covered by the guideline is (are) specifically described.	7	7	3	7	7
3. The population (patients, public, etc.) to whom the guideline is meant to apply is specifically described.	7	7	7	7	7
**DOMAIN 2. STAKEHOLDER ENGAGEMENT**					
4. The guideline development group includes individuals from all relevant professional groups.	7	7	1	1	7
5. The views and preferences of the target population (patients, public, etc.) have been sought.	7	7	1	1	7
6. The target users of the guideline are clearly defined.	7	7	3	3	7
**DOMAIN 3. RIGOUR OF DEVELOPMENT**					
7. Systematic methods were used to search for evidence.	7	7	3	7	7
8. The criteria for selecting the evidence are clearly described.	7	7	7	7	7
9. The strengths and limitations of the body of evidence are clearly described.	7	7	6	7	7
10. The methods for formulating the recommendations are clearly described.	7	7	7	7	7
11. The health benefits, side effects, and risks have been considered in formulating the recommendations.	7	7	7	7	7
12. There is an explicit link between the recommendations and the supporting evidence.	7	7	7	7	7
13. The guideline has been externally reviewed by experts prior to its publication.	7	7	7	7	7
14. A procedure for updating the guideline is provided.	7	5	1	7	7
**DOMAIN 4. CLARITY OF PRESENTATION**					
15. The recommendations are specific and unambiguous.	7	7	7	7	7
16. The different options for management of the condition or health issue are clearly presented.	7	7	7	7	7
17. Key recommendations are easily identifiable.	7	7	7	7	7
**DOMAIN 5. APPLICABILITY**					
18. The guideline describes facilitators and barriers to its application.	7	7	7	5	7
19. The guideline provides advice and/or tools on how the recommendations can be put into practice.	7	7	7	7	7
20. The potential resource implications of applying the recommendations have been considered.	5	7	6	5	7
21. The guideline presents monitoring and/or auditing criteria.	7	7	6	7	7
**DOMAIN 6. EDITORIAL INDEPENDENCE**					
22. The views of the funding body have not influenced the content of the guideline.	7	7	7	7	7
23. Competing interests of guideline development group members have been recorded and addressed.	7	7	7	7	7
**OVERALL GUIDELINE ASSESSMENT**					
**Rate the overall quality of this guideline.**	**6.9**	**6.9**	**5.4**	**6.1**	**7.0**
**I would recommend this guideline for use.**	**Yes**	**Yes**	**Yes**	**Yes**	**Yes**

Comments/Notes

- AUA—Procedure for updating included in 2022 revision.
- BSUG/RCOG—Indicates first iteration of guideline; however, schedule for updating not detailed.
- CUA—Lacks introduction providing overview of scope and purpose. Stakeholder engagement does not mention inclusion of allied health professionals and/or patients/public in the development process.
- EAG—Stakeholder engagement does not mention inclusion of interprofessional team or patients/public in guideline development process.
- EUA— Stakeholder engagement does not mention inclusion of interprofessional team or patients/public in guideline development process

AUA = American Urological Association
BSUG/RCOG = British Society of Urogynaecology/Royal College of Obstetricians and Gynaecologists
CUA = Canadian Urological Association
EAG = East Asian group of urologists
EUA = European Association of Urology

Ranked on Scale of 1 to 7
1 = Strongly Disagree
7 = Strongly Agree

Table S3: **IC/BPS Part 2**—Summary of Agree II Quality Analysis of Clinical Guidelines

	Quality Score			
	**GIBS**	**ICS**	**ISPOG**	**JCS**
**DOMAIN 1. SCOPE AND PURPOSE**				
1. The overall objective(s) of the guideline is (are) specifically described.	7	7	7	7
2. The health question(s) covered by the guideline is (are) specifically described.	7	7	7	7
3. The population (patients, public, etc.) to whom the guideline is meant to apply is specifically described.	7	7	7	7
**DOMAIN 2. STAKEHOLDER ENGAGEMENT**				
4. The guideline development group includes individuals from all relevant professional groups.	5	7	3	7
5. The views and preferences of the target population (patients, public, etc.) have been sought.	2	7	3	7
6. The target users of the guideline are clearly defined.	7	7	7	7
**DOMAIN 3. RIGOUR OF DEVELOPMENT**				
7. Systematic methods were used to search for evidence.	2	7	7	7
8. The criteria for selecting the evidence are clearly described.	2	7	7	7
9. The strengths and limitations of the body of evidence are clearly described.	2	7	7	7
10. The methods for formulating the recommendations are clearly described.	2	7	3	7
11. The health benefits, side effects, and risks have been considered in formulating the recommendations.	7	7	7	7
12. There is an explicit link between the recommendations and the supporting evidence.	7	7	5	7
13. The guideline has been externally reviewed by experts prior to its publication.	2	7	7	7
14. A procedure for updating the guideline is provided.	7	7	5	7
**DOMAIN 4. CLARITY OF PRESENTATION**				
15. The recommendations are specific and unambiguous.	7	7	7	7
16. The different options for management of the condition or health issue are clearly presented.	7	7	7	7
17. Key recommendations are easily identifiable.	7	7	7	7
**DOMAIN 5. APPLICABILITY**				
18. The guideline describes facilitators and barriers to its application.	7	7	5	7
19. The guideline provides advice and/or tools on how the recommendations can be put into practice.	7	7	7	7
20. The potential resource implications of applying the recommendations have been considered.	7	7	6	7
21. The guideline presents monitoring and/or auditing criteria.	7	7	7	7
**DOMAIN 6. EDITORIAL INDEPENDENCE**				
22. The views of the funding body have not influenced the content of the guideline.	4	7	7	7
23. Competing interests of guideline development group members have been recorded and addressed.	1	7	7	7
**OVERALL GUIDELINE ASSESSMENT**				
**Rate the overall quality of this guideline.**	**5.2**	**7.0**	**6.2**	**7.0**
**I would recommend this guideline for use.**	**Yes**[a]	**Yes**	**Yes**[b]	**Yes**

Comments/Notes

- GIBS—Stakeholder engagement does not mention inclusion of interprofessional team or patients/public in guideline development process. Sponsor product promotions could have also weakened editorial independence.
- ICS—Format is collection of journal articles and other documents, thus some variations in methodologies employed for guideline development.
- ISPOG—Short, published version used in analysis; long version was not published and thus is not publicly available.
- JCS—Little details about specifics relating to ICS/BPS included in guidelines, and no patients in guideline development or review of the guideline.

a. Yes, with modifications: Modifications recommended are the removal of advertising and inclusion of confirmational statement that funder(s) of guideline did not influence content of the guideline.
b. Yes, depending upon the long version of the guideline: If the long version addresses limitations of the short version, this guideline offers sound guidance.

GIBS = Global IC/PBS Society
ICS = International Continence Society
ISPOG = International Society of Psychosomatic Obstetrics and Gynecology
JCS = Japanese Continence Society

Ranked on Scale of 1 to 7
1 = Strongly Disagree
7 = Strongly Agree

Table S4: Overactive Bladder Part 1—Summary of Agree II Quality Analysis of Clinical Guidelines

	Quality Rating						
	**ACOG AUGS**	**AUA SUFU**	**CUA**	**EUA**	**ICS**	**JCS**	**SANZ UGSA**
**DOMAIN 1. SCOPE AND PURPOSE**							
1. The overall objective(s) of the guideline is (are) specifically described.	7	7	7	7	7	7	7
2. The health question(s) covered by the guideline is (are) specifically described.	7	7	7	7	7	7	7
3. The population (patients, public, etc.) to whom the guideline is meant to apply is specifically described.	7	7	7	7	7	7	7
**DOMAIN 2. STAKEHOLDER ENGAGEMENT**							
4. The guideline development group includes individuals from all relevant professional groups.	5	7	3	7	7	7	6
5. The views and preferences of the target population (patients, public, etc.) have been sought.	1	2	2	7	7	7	2
6. The target users of the guideline are clearly defined.	7	7	7	7	7	7	7
**DOMAIN 3. RIGOUR OF DEVELOPMENT**							
7. Systematic methods were used to search for evidence.	5	7	7	7	7	7	7
8. The criteria for selecting the evidence are clearly described.	7	7	7	7	7	7	2
9. The strengths and limitations of the body of evidence are clearly described.	7	7	7	7	7	7	5
10. The methods for formulating the recommendations are clearly described.	7	7	7	7	7	7	4
11. The health benefits, side effects, and risks have been considered in formulating the recommendations.	7	7	7	7	7	7	7
12. There is an explicit link between the recommendations and the supporting evidence.	7	7	7	7	7	7	7
13. The guideline has been externally reviewed by experts prior to its publication.	7	7	7	7	7	7	7
14. A procedure for updating the guideline is provided.	7	7	4	7	7	7	7
**DOMAIN 4. CLARITY OF PRESENTATION**							
15. The recommendations are specific and unambiguous.	7	7	7	7	7	7	7
16. The different options for management of the condition or health issue are clearly presented.	7	7	7	7	7	7	7
17. Key recommendations are easily identifiable.	7	7	7	7	7	7	7
**DOMAIN 5. APPLICABILITY**							
18. The guideline describes facilitators and barriers to its application.	7	7	7	7	7	7	7
19. The guideline provides advice and/or tools on how the recommendations can be put into practice.	7	7	7	7	7	7	7
20. The potential resource implications of applying the recommendations have been considered.	7	7	7	7	7	7	5
21. The guideline presents monitoring and/or auditing criteria.	7	6	7	7	7	7	5
**DOMAIN 6. EDITORIAL INDEPENDENCE**							
22. The views of the funding body have not influenced the content of the guideline.	7	7	7	7	7	7	7
23. Competing interests of guideline development group members have been recorded and addressed.	7	7	7	7	7	7	1
**OVERALL GUIDELINE ASSESSMENT**							
Rate the overall quality of this guideline.	**7.0**	**7.0**	**6.5**	**7.0**	**7.0**	**7.0**	**5.9**
I would recommend this guideline for use.	**Yes**	**Yes**	**Yes**	**Yes**	**Yes**	**Yes**	**Yes**

Comments/Notes

- ACOG/AUGS—No mention of inclusion of interprofessional team or patients in guideline development or review of the guideline.
- AUA/SUFU—No mention of inclusion of patients in guideline development or review of the guideline.
- CUA—No mention of inclusion of interprofessional team or patients in guideline development or review of the guideline.
- EAU— Stakeholder engagement does not mention inclusion of interprofessional team or patients/public in guideline development process.
- ICS—Document presents standard terminology but offers expert insights into condition.
- JCS—No patients in guideline development or review of the guideline.
- SANZ/UGSA—No grading schema employed to clarify strength of evidence for recommendations and did not list author disclosures.

ACOG = American College of Obstetricians and Gynecologists
AUGS = American Urological Association
AUA = American Urological Association
CUA = Canadian Urological Association
EAU – European Association of Urology
ICS = International Continence Society
JCS = Japanese Continence Society
SANZ = Urological Society of Australia and New Zealand
UGSA = Urogynaecological Society of Australia SUFU = Society of Urodynamics, Female Pelvic Medicine & Urogenital Reconstruction

Ranked on Scale of 1 to 7
1 = Strongly Disagree
7 = Strongly Agree

Table S5: Stress Urinary Incontinence—Summary of Agree II Quality Analysis of Clinical Guidelines

	Quality Rating					
	**ACOG AUGS**	**AUA SUFU**	**EAU**	**ICS IUGA**	**JCS**	**SOGC**
**DOMAIN 1. SCOPE AND PURPOSE**						
1. The overall objective(s) of the guideline is (are) specifically described.	7	7	7	7	7	7
2. The health question(s) covered by the guideline is (are) specifically described.	7	7	7	7	7	7
3. The population (patients, public, etc.) to whom the guideline is meant to apply is specifically described.	7	7	7	7	7	7
**DOMAIN 2. STAKEHOLDER ENGAGEMENT**						
4. The guideline development group includes individuals from all relevant professional groups.	5	5	7	7	7	7
5. The views and preferences of the target population (patients, public, etc.) have been sought.	1	7	7	7	7	5
6. The target users of the guideline are clearly defined.	7	7	7	7	7	7
**DOMAIN 3. RIGOUR OF DEVELOPMENT**						
7. Systematic methods were used to search for evidence.	7	7	7	7	7	7
8. The criteria for selecting the evidence are clearly described.	5	7	7	7	7	7
9. The strengths and limitations of the body of evidence are clearly described.	7	7	7	7	7	7
10. The methods for formulating the recommendations are clearly described.	5	7	7	7	7	7
11. The health benefits, side effects, and risks have been considered in formulating the recommendations The health benefits, side effects, and risks have been considered in formulating the recommendations.	7	7	7	7	7	7
12. There is an explicit link between the recommendations and the supporting evidence.	7	7	7	7	7	7
13. The guideline has been externally reviewed by experts prior to its publication.	7	7	7	7	7	7
14. A procedure for updating the guideline is provided.	7	6	7	7	7	7
**DOMAIN 4. CLARITY OF PRESENTATION**						
15. The recommendations are specific and unambiguous.	7	7	7	7	7	7
16. The different options for management of the condition or health issue are clearly presented.	7	7	7	7	7	7
17. Key recommendations are easily identifiable.	7	7	7	7	7	7
**DOMAIN 5. APPLICABILITY7**						
18. The guideline describes facilitators and barriers to its application.	7	7	7	7	7	7
19. The guideline provides advice and/or tools on how the recommendations can be put into practice.	7	7	7	7	7	7
20. The potential resource implications of applying the recommendations have been considered.	7	7	7	7	7	7
21. The guideline presents monitoring and/or auditing criteria.	7	7	7	7	7	7
**DOMAIN 6. EDITORIAL INDEPENDENCE**						
22. The views of the funding body have not influenced the content of the guideline.	7	7	7	7	7	7
23. Competing interests of guideline development group members have been recorded and addressed.	7	7	7	7	7	7
**OVERALL GUIDELINE ASSESSMENT**						
Rate the overall quality of this guideline.	**7.0**	**7.0**	**7.0**	**7.0**	**7.0**	**7.0**
I would recommend this guideline for use.	**Yes**	**Yes**	**Yes**	**Yes**	**Yes**	**Yes**

Comments/Notes

- ACOG/AUGS—No mention of inclusion of interprofessional team or patients in guideline development or review of the guideline.
- AUA/SUFU—No mention of inclusion of interprofessional team or patients in guidelines development process.
- EAU— Stakeholder engagement does not mention inclusion of interprofessional team or patients/public in guideline development process.
- ICS/IUGA—Format is collection of journal articles and other documents, thus some variations in methodologies employed for guideline development.
- JCS—No patients in guideline development or review of the guideline.
- SOGC—Employed evidence grading system; however, nutrition recommendations not scored.

ACOG = American College of Obstetricians and Gynecologists
AUGS = American Urological Association
AUA = American Urological Association
EAU – European Association of Urology
ICS = International Continence Society
IUGA = International Urogynecological Association
JCS = Japanese Continence Society
SOGC = Society of Obstetricians and Gynaecologists of Canada
SUFU = Society of Urodynamics, Female Pelvic Medicine & Urogenital Reconstruction

Ranked on Scale of 1 to 7
1 = Strongly Disagree
7 = Strongly Agree
